# A morphological study of the shape of the corpus callosum in normal, schizophrenic and bipolar patients

**DOI:** 10.1111/joa.13777

**Published:** 2022-10-13

**Authors:** Christiaan L. Vermeulen, Peet J. du Toit, Gerda Venter, Rene Human‐Baron

**Affiliations:** ^1^ Department of Physiology, School of Medicine, Faculty of Health Sciences University of Pretoria Pretoria South Africa; ^2^ Associate of the Institute for Food, Nutrition and Wellbeing University of Pretoria Pretoria South Africa; ^3^ Associate of the Institute for Cellular and Molecular Medicine University of Pretoria Pretoria South Africa; ^4^ Associate of the Exercise Smart Team University of Pretoria Pretoria South Africa; ^5^ Department of Anatomy, School of Medicine, Faculty of Health Sciences University of Pretoria Pretoria South Africa

**Keywords:** Corpus callosum, landmarks, morphology

## Abstract

Abnormalities in the morphology of the corpus callosum have been found to be involved in cognitive impairments or abnormal behaviour in patients with mental disorders such as schizophrenia and bipolar disorder. The present study investigated morphological shape differences of the corpus callosum in a large cohort of 223 participants between normal, schizophrenic and bipolar patients on MRI scans, CT scans and cadaver samples. Healthy samples were compared to a mental disorder population sample to determine morphological shapes variations associated with schizophrenia and bipolar disorder. Landmark‐based methodology was used to contour the corpus callosum shape that served as standard positions to allow for radial and thickness partitioning in order to determine shape variations within the specific localised anatomical sections of the corpus callosum. Shape analysis was performed using Ordinary Procrustes averaging and superimposing landmarks to define an average landmark position for the specific regions of the corpus callosum. No significant global shape differences were found between the different mental disorders. Schizophrenia and bipolar shapes differed mostly in the genu‐rostrum, posterior body, isthmus and splenium. Sample group comparisons yielded significant differences between all groups and global measurement parameters and in various sub‐regions. The findings of the present study suggest that the corpus callosum in schizophrenia and bipolar differs significantly compared to healthy controls, specifically in the anterior body and isthmus for schizophrenia and only in the isthmus for bipolar disorder. Shape changes in these regions may possibly, in part, be responsible for the symptoms and cognitive impairments observed in schizophrenia and bipolar disorder.

## Graphical abstract

The corpus callosum in schizophrenia and bipolar differs compared to healthy controls, specifically in the anterior body and isthmus which interconnect the prefrontal, temporal and parietal regions.

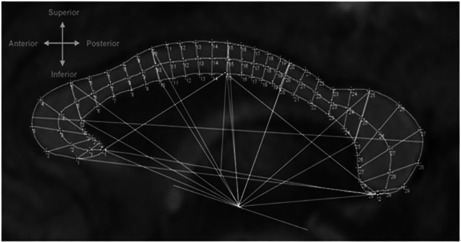

## 1 INTRODUCTION

The corpus callosum (CC) is a cerebral structure found in the brain consisting of bundles of axonal nerve fibres that transmit information between the two cerebral hemispheres (Downhill et al., 2000; Luders et al., 2010; Ozdemir et al., 2007; Snell, 2010). It has been shown to have significant critical functions in interhemispheric transfer of information in the brain as opposed to previous beliefs of serving only little function, mainly only anatomical functioning (Myers & Sperry, 1958). Modern split‐brain studies, patients undergoing complete or partial callostomies, as well as callosal lesion studies have greatly contributed to further insights on the morphology, structure and function of the CC (Gazzaniga, 2005; Glickstein, 1995).

The insights from past CC studies and advances in neuroimaging technology have allowed for more in‐depth investigation of mental disorders and their association with macro‐ and micro‐morphological changes and regional neuropathology, specifically in the CC that are undetectable by the naked eye (Whitford et al., 2010). Morphological abnormalities of the CC have been noted in schizophrenia, bipolar disorder, depression, personality disorders, autism, attention deficit‐hyperactivity disorder and phantom limb syndrome to name a few (Van der Knaap & van der Ham, 2011).

Numerous mental disorders share similar characteristics and symptoms making an accurate diagnosis difficult in some cases. However, each mental disorder presents a distinct combination of symptoms and characteristics, which should align to its specific diagnostic criteria in the Diagnostic and Statistical Manual of Mental Disorders 5th Edition (DSM‐5), to be accurately diagnosed. In the present study mental disorders, specifically schizophrenia and bipolar disorder, are highlighted.

### 1.1 Schizophrenia

Schizophrenia is a severe clinical cognitive disorder with a life‐time risk of 0.5%–1% (Andreasen & Carpenter Jr, 1993; Consortium, 2009). The disorder manifests early in life and is involved in the degradation and impairment of the relationships between thought, emotions and behaviour. It is characterised by dissolution and withdrawal from reality and social interaction, delusions, hallucinations (mostly auditory) and severe problematic cognition (Consortium, 2009).

These symptoms and behaviours exhibited by schizophrenics indicate dysfunction of connections in cortical regions of the brain that transfer information through the CC or dysfunction in the CC to transfer information to cortical regions. The CC is seen as a primary causal structure for the disorder (David, 1994; Van der Knaap & van der Ham, 2011). AS David (1994) suggests the disorder could be a result of hyper‐connectivity in the CC. An excessive amount of information is transferred to the target cortical region creating interference or conflict in the processing and integration of the information.

Morphological changes in CC shape and size may influence the transfer and integration of information causing CC dysfunction. Disparities in research regarding CC shape and size have largely been due to study limitations such as small sample sizes and skewed demographics; sample integrity; outdated neuroimaging technologies; non‐uniformity in landmark identification and different methodologies of measurement used (Downhill et al., 2000).

The findings in the literature indicate either an increased or decreased CC size or thickness, variations in shape and decreased area dimensions in cross‐sectional measurements. Furthermore, decreased fibre density in specific regions of the CC has been seen from the anterior section right through to the posterior section in post‐mortem (cadaver) studies of individuals with clinical diagnosis of schizophrenia compared to normal controls (Arnone, McIntosh, Tan, & Ebmeier, 2008).

Although MRI studies have contributed to the research approach towards better insights there are still shortfalls, posing inconsistent results on CC morphological dimension analysis and measurements. The general consensus points to variations in CC shape and decreased CC size and area in schizophrenics as opposed to healthy controls (Arnone, McIntosh, Tan, & Ebmeier, 2008; Downhill et al., 2000; Mitelman et al., 2009).

### 1.2 Bipolar mood disorder

Bipolar disorder is a mood disorder characterised by periods of mania and depression resulting in inconsistent emotional responses to stimuli. The disorder portrays an instability between emotion, thought and behaviours). Bipolar disorder has been found to indicate abnormalities found in the white matter tracts, having a primary focus on the CC (Sarrazin et al., 2015). In the DSM‐5, bipolar disorder has been considered as a bridge between the schizophrenia spectrum and other psychotic disorders due to the similarities in the clinical presentations of the disease (Li et al., 2014).

The similar clinical presentation of bipolar disorder and schizophrenia has caused an increase in investigations aimed at discovering possible morphological changes and neuropathology's of the CC that may pose a cause of this disorder. A meta‐analysis of the CC in bipolar disorder found significantly reduced CC area indicating problematic interhemispheric transfer of information (Arnone, McIntosh, Chandra, & Ebmeier, 2008). Recently Sarrazin et al. (2015) found similar results where the area in the posterior sections (mid‐body, isthmus and splenium) of the CC is reduced. Li et al. (2014) found reduced white matter fibre integrity in the posterior sections (anterior genu, posterior genu, posterior body and anterior splenium) of the CC.

The abovementioned studies reiterate the involvement of the CC playing a critical role in the manifestations of the behavioural symptoms observed in schizophrenia and bipolar disorder (Downhill et al., 2000; Francis et al., 2016; Funnell et al., 2000; Prendergast et al., 2015; Prendergast et al., 2018; Walterfang et al., 2008).

Significant findings regarding the variations in the morphology of the CC are still lacking (Francis et al., 2016). In fact, the literature indicates conflicting findings regarding the variations of CC in patients and even in normal individuals. Numerous studies (Arnone, McIntosh, Chandra, & Ebmeier, 2008; Arnone, McIntosh, Tan, & Ebmeier, 2008; Constant & Ruther, 1996; Davatzikos & Resnick, 1998; Ozdemir et al., 2007) have reported that these differences in the shape and size of the CC are either due to normal variations, age and sex or psychological disorders and neurodegenerative disease factors.

Most CC morphology studies have used quantitative morphological assessment of the CC (length, width, height, area and volume) to equate the variations in normal and patient samples (Ozdemir et al., 2007). This type of analysis does not account for minor structural or shape variations at specific locations in the CC, especially volume and area measurements (Ozdemir et al., 2007). The shape of a biological structure is also a very difficult parameter to measure as there are not many descriptors for shape. Normally, the shape of an object or structure is described in terms of meaning and understanding, comparing the shape to an already existing known shape. An example of this would be “the splenium of the CC is bulbous” indicating a round shape as opposed to “the splenium of the CC is tubular” indicating a narrow tube shape. This makes it difficult to report on the observed shape of the CC as each researcher might have a different meaning, definition or interpretation of an existing known shape (Russ, 2006).

Therefore, landmarks that capture small variations in shape can quantify descriptors to analyse the CC morphology (Bookstein, 1997; Downhill et al., 2000; Joshi et al., 2013).

The aim of the present study was to investigate morphological shape differences of the CC between normal, schizophrenic and bipolar patients from MRI scans, CT scans and cadaver samples of a South African population. Healthy population samples (MRI scans and cadaver brains) were compared to a mental disorder population sample (CT scans) in order to obtain comprehensive data on CC shape differences in an attempt to account for inconsistencies noted in the literature and determine potential morphological shapes variations associated with schizophrenia and bipolar disorder to aid in the disorder diagnostic process. Intra‐ and interobserver error measures addressed consistency, reliability and accuracy of the results obtained in this study.

Thus, the objectives of this study were to investigate the shape of the CC by taking measurements, using digital software on cadaveric brains, MRI and CT scans to have a holistic approach in identifying shape inconsistencies between different scan modalities, investigate and define a reliable, reproducible set of landmarks to determine accurate and reliable anatomical coordinates, investigate whether the shape of the CC is affected by schizophrenia and bipolar disorder. This was done by comparing the results of this study to the literature.

## 2 METHODS AND MATERIALS

The total sample (*n* = 223) consisted of three smaller individual sample groups: a cadaveric sample, an MRI sample and a CT‐scan sample. The cadaver sample population comprised 50 harvested brain specimens which were cut midsagitally using a brain knife. One hemisphere of each cadaver brain was examined and recorded by means of a digital photograph. The cadaver brain sample was further subdivided according to sex and consisted of 29 males and 21 females. MRI scans were acquired using a GE Signa 1.5 T MRI (T_E_ = 14 ms, T_R_ = 466 ms, Thickness = 5 mm). The MRI population was comprised 111 patients' scans. The MRI scan sample was further subdivided according to sex and consisted of 50 males and 61 females. The MRI scans were classified as healthy or normal by the Radiologists on duty when the scans were conducted on patients. CT scans were acquired using a 16‐section multidetector CT scanner. The scanning parameters were as follow: acquisition 12 × 0.6 mm, rotation time 1 s, pitch 0.8, 120 kV tube voltage. Reconstruction parameters were 0.3 mm reconstruction increment, 90 mm reconstruction FOV and U90u kernel. The CT scans were composed of 62 patient scans. The CT scan sample was further subdivided according to sex and consisted of 41 males and 21 females. The CT scans were classified at the respective institutions by the Radiologists on duty when the scans were conducted on patients. However, the CT scans were classified, not only by the Radiologists but also by the prescribed Psychiatrist assigned to the specific patient confirming pathology in the patient.

Study participants that were 18 years and older were included in the study with no limitations to ethnicity, weight and height. Participants were, however, excluded from the healthy cadaver and MRI sample if there was known or visible pathology to the cerebral hemispheres; surgery to the cerebral hemispheres or poorly embalmed/scan resolutions. Participants were excluded from the mental disorder CT sample if there was known or visible pathology to the cerebral hemispheres (other than associated with schizophrenia, bipolar disorder and psychosis); surgery to the cerebral hemispheres or poor scan resolutions.

### 2.1 Measurements

In the present study, global and local analysis was performed on all three sample types to address the study objectives. Landmark‐based methodology as seen in (Downhill et al., 2000) was used to contour the CC shape and account for slight shape variations. The CC landmarks served as standard positions to allow for radial partitioning and thickness partitioning methods to be performed in order to determine shape variations within the specific localised anatomical sections of the CC.

In the present study, a combination of numerical size descriptors (length, width and area) as well as established shape descriptors (roundness, curvature and angle) were used to describe the shape of the CC. ImageJ software (Rasband, 2018) was used to conduct the measurements. The software allowed for increased measurement sensitivity, accuracy and image standardisation through image magnification to reach shape contour boundary pixelation. The CC was scaled/adjusted in accordance to brain size (most anterior to most posterior point of the midsagittal brain) and the measurement units were calibrated according to the scale present on the digital cadaver photographs as well as the scale presented on the MRI and CT scans. A bounding box was inserted with a width of 120 mm and a height of 60 mm. The scan was cropped according to the dimensions of the bounding box which was at a magnification of 400% to account for accuracy and image orientation standardisation.

All measurement units were in millimetres (mm) and the measurement parameters used to analyse the shape of the CC are shown in Table 1.

**TABLE 1:** CC measurement parameters

CC shape measurement parameter abbreviation	CC shape measurement parameter	Parameter description	Measurement (mm): Type Description
Image preparation			
	Scale	CC scaled and measurements calibrated according to each individual image scale present on the image	Straight line Length
	Bounding Box	Width of 120 mm and a height of 60 mm	Straight line Length & width
Measurement parameters			
OT	Outline trace	Full CC perimeter traced	Polygon Area & roundness
APIL	Anterior—posterior inferior line	Straight line from the most inferior border of the rostrum and splenium	3 Straight line 4 Length
TH	Total height	Distance from the most superior point of the CC to where it meets the APIL	Straight line Length
TL	Total length	Distance from the most anterior point to the most posterior point of the CC	Straight line Length
GW	Genu region width	Distance from the superior perimeter to the inferior perimeter of the CC genu	Straight line Width
ABW	Anterior‐body region width	Distance from the superior perimeter to the inferior perimeter of the CC anterior‐body	Straight line Width
PBW	Posterior‐body region width	Distance from the superior perimeter to the inferior perimeter of the CC mid‐body	Straight line Width
ISW	Isthmus width	Distance from the superior perimeter to the inferior perimeter of the CC Posterior‐body	Straight line Width
ALM	Most anterior landmark	Landmark position at the posterior tip of the genu‐rostrum	Landmark X Y Coordinates
PLM	Most posterior‐inferior landmark	Landmark position at the most inferior tip of the splenium	Landmark X Y Coordinates
SLM1‐29	Superior landmark 1–29	29 Evenly spaced landmarks on the superior CC perimeter between the posterior tip of the rostrum and the most inferior tip of the splenium	Landmark X Y Coordinates
ILM1‐29	Inferior landmark 1–29	29 Evenly spaced landmarks on the inferior CC perimeter between the posterior tip of the rostrum and the most inferior tip of the splenium	Landmark X Y Coordinates
WLM1‐29	Width landmark 1–29 (29 partitioning lines)	Distance between the superior and inferior corresponding perimeter landmarks (1–29) of the CC	Straight line Width
SC	Spline curve	Plotting of a curvilinear line by equally dividing each of the 29 partitioning lines of the CC	Polygon Area & roundness
CLM1‐29	Centre landmark 1–29	29 central landmarks positioned at the intersection of the curvilinear line and the partitioning lines of the CC	Landmark X Y Coordinates
APC	AC‐PC Line centre	Distance from the superior edge of the anterior commissure to the inferior edge of the posterior commissure equally divided	Straight line Length
ALM‐APC	Anterior landmark to AC‐PC centre	Distance from the most anterior landmark to the AC‐PC centre	Straight line Length
ILM (5, 10,15, 20, 25) ‐APC	Inferior landmark 5, 10, 15, 20, 25 to AC‐PC centre	Distance from inferior landmarks 5, 10, 15, 20, 25 to the AC‐PC centre	Straight line Length
PLM‐APC	Posterior landmark to AC‐PC centre	Distance from the most posterior landmark to the AC‐PC centre	Straight line Length
SLM (5, 10,15, 20, 25)‐APC	Superior landmark 5 to AC‐PC centre	Distance from superior landmarks 5, 10, 15, 20, 25 to the AC‐PC centre	Straight line Length
ANGLE	CC angle	Angle between most anterior landmark, inferior landmark 15 and most posterior landmark	Angle Degrees

The CC outline (OT) was traced according to the outline shown in the midsagittal sections of the digital cadaver photographs, MRI and CT scans. Points were placed on the exact pixel edges, based on the grey‐scale value differences, between the CC and the surrounding regions to determine the rough polygon shape of the CC. The polygon shape was interpolated according to pixel grey‐scale values to produce a smooth outline for additional measurement accuracy and minimal sensitivity to shape orientation of the CC. Fitting of the OT allowed for CC shape analyses of roundness and area. The software computed the roundness automatically by algorithm using the following formula: (4 × ([𝐴𝑟𝑒𝑎])/(𝜋 × 〖[𝑀𝑎𝑗𝑜𝑟 𝐴𝑥𝑖𝑠]〗^2^))

The CC was subdivided into anatomical sections in an anterior to posterior direction: genu‐rostrum, anterior body, mid‐body, posterior body, isthmus and splenium. The distances of the partitioning lines were measured from the superior to inferior OT to determine the width of the genu (GW), anterior body (ABW), posterior body (PBW) and isthmus (ISW). The partitioning lines were drawn in proportion to the overall CC shape contour of each individual CC and not by 90‐degree vertical lines. The partitioning lines indicated the start and‐or end locus of each anatomical section of the CC.

Landmark points were positioned at the posterior tip of the rostrum (ALM) and the most posterior‐inferior tip of the splenium (PLM) by the selection of the multi‐point tool. Twenty‐nine landmarks were evenly plotted between the ALM and the PLM on the superior OT (SLM1‐29) as well as the inferior OT (ILM1‐29) in proportion to the observed shape and anatomical partitioned sections of the CC. Four landmarks were plotted evenly on the superior OT as well as the inferior OT of each anatomical section. Another landmark was plotted at each partitioning line and OT intersection and used as reference landmark positions (SLM 5, 10, 15, 20, 25 and ILM 5, 10, 15, 20, 25) for the specific CC anatomical section start and end coordinates.

The distance between each of the 29 superior and inferior landmarks was measured with a line (WLM1‐29) to the opposite corresponding landmark position. The distance of the connected lines was measured to determine the width of the CC within each anatomical section. The measured WLM1‐29 distances were divided equally to define the centre positions. A spline curve or curvilinear was fitted by selecting the polygon tool to plot the spline coordinates at the exact centre positions, starting at the ALM and including the PLM.

**FIGURE 1:**
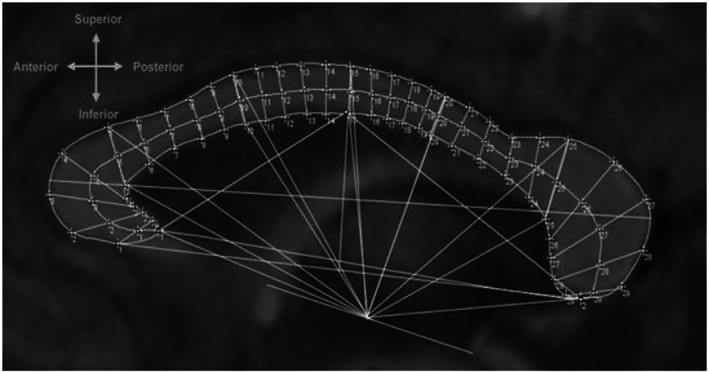
Method of partitioning and measuring the shape of the CC.

A line was drawn from the superior edge of the anterior commissure to the inferior edge of the posterior commissure known as the AC‐PC line used in brain scan analysis due to its defined location (Cox, 1996). The distance of the AC‐PC line was divided equally to define the centre position (APC). The distances between the CC section landmarks SLM 5, 10,15, 20, 25 and ILM 5, 10,15, 20, 25, were measured to the APC as well as the distances between the ALM and PLM landmarks were measured to the APC. The above measurements were referred to as the SLM 5, 10,15, 20, 25‐APC; ILM 5, 10,15, 20, 25‐APC; ALM‐APC and PLM‐APC lines. An additional angle measurement was made, by selecting the angle tool, between the ALM and PLM, using ILM 15 as the angle pivot point (vertex angle). Figure 1 shows the method of partitioning and measuring the shape of the CC.

### 2.2 Statistical analysis

The descriptive statistics including the minimum, maximum, mean, standard deviation and inter‐quartile range were used to describe the morphological measurements made of the CC. The descriptive statistics were also used to describe the minimum, maximum, mean, standard deviation and inter‐quartile range between sex and age of the normal control samples and patient samples. From each data sample set (MRI, CT and Cadaver), 30 intra‐ and inter‐rater measurements were conducted at random selection to account for accuracy and reliability of the methodology and the results obtained from this study. Intra‐ and inter‐rater agreement was determined using the intraclass correlation coefficient (two‐way mixed, absolute agreement).

ANOVA was used to compare means of outcome variables measured on images between groups as defined by imaging samples (MRI, CT, Cadaver) or disorders. Appropriate post hoc tests were applied to determine which groups differ significantly. ANCOVA was used to adjust for age. A Kruskal–Wallis was performed when outliers were excluded as sensitivity analyses. Where multiple comparisons were run in the cases where subgroups were compared (e.g., MRI vs. CT or Bipolar vs. Schizophrenic), statistical significance was taken at a 1% level in order to be conservative and not obtain too many false significant differences. Where only two groups were compared the t‐test or Mann–Whitney's U test was used as appropriate. Spearman's Roh correlations were calculated as measure of association between continuous outcomes. Non‐parametric statistical analyses were used due to comparisons within sample groups that were small and the assumption of normal distribution may be too strong in many of these comparisons.

The measurement data from the 62 CT scans were used as the experimental group which was compared to the normal group data from the 50 cadaver cerebral photographs and 111 MRI scans. The comparisons were based on two‐way ANOVA cell means, anteroposterior, of 82 females in the normal group and 21 in the experimental group as well as 79 males in the normal group and 41 in the experimental group.

Ordinary Procrustes analysis (OPA) was conducted in order to compare the shapes of different groups to one another. Before the OPA was conducted, the median coordinate pairs for each of the 60 landmarks (29 superior landmarks and the most anterior landmark as well as 29 inferior landmarks and the most posterior landmark) were calculated for the relevant groups (refer to Table 1). These were used for the OPA. Comparisons were run for different groups, that is the MRI, CT and Cadaver samples. Furthermore, the different disorders within the CT sample were compared with one another, as well as the disorders against the MRI. In order to compare the shapes statistically, the x–y coordinate pairs of the OPA‐adjusted shapes were compared using Hotelling's *T*‐squared test for comparing bivariate means. Due to shapes being centred around the origin, sections were compared with one another. This introduces multiplicity and allows for the reporting of the p‐value from Hotelling's *T*‐squared test, as well as a *p*‐value multiplied by three and multiplied by six. This provides a robust perspective on the differences. Statistical significance for Hotelling's T‐squared test was taken at a 5% level. The OPA was conducted using the R package shapes.

All statistical analysis was done using STATA statistical software, version 14, developed by StataCorp LP in 2015.

## 3 RESULTS

### 3.1 Cadaver sample results

Measurements were performed on 50 cadaver brains specimens. The ages of six individuals in the sample were unknown, resulting in the exclusion of these individuals from age comparisons. These individuals were, however, included in comparisons related to sample groups (refer to A1).

### 3.2 MRI sample results

Measurements were performed on 111 healthy MRI scans (refer to A2).

### 3.3 CT sample results

Measurements were conducted on 62 CT scans of patients with various mental disorders. The CT scans of patients were used in the experimental group. The CT sample was, subdivided according to the various diagnosed mental disorders associated with each patient's CT scan. Some of the patients in the CT sample were not diagnosed with a specific mental disorder when the scans were conducted and showed symptoms of various mental disorders, however, these patients were still included in the sample indicated by “other mental disorders”. The mental disorder of one patient in the sample was unknown, resulting in the exclusion of the individual from sample group comparisons (refer to A3).

Kruskal–Wallis test results showed no significant differences (*p* > 0.001) for any of the CC shape parameters measured between all the mental disorders. The *p*‐values ranged from 0.002 to 0.9513. Although there were no statistically significant differences, the widths measured between the inferior and superior landmark 26 and 27 (WLM26 and WLM27) were just above the level of significance with *p*‐values of 0.002 and 0.008, respectively.

Due to the findings from the Kruskal–Wallis test showing some of the parameters being just above the level of significance, a post hoc test was conducted to determine which of the mental disorders differed from one another within the CT sample, therefore, a bivariate comparison was performed using the Mann–Whitney U test for each measurement parameter (refer to A4). The findings from the analysis showed no significant differences (*p* > 0.001), however, for the length measured between the ALM and APC for the comparison between bipolar and psychosis, the p‐value was just above the level of significance (*p* = 0.009). This was also observed in the WLM8 (*p* = 0.006), WLM26 (*p* = 0.003), WLM27 (*p* = 0.002), WLM28 (*p* = 0.006) and WLM29 (*p* = 0.005) measurement parameters for the comparison between the other mental disorders and schizophrenia.

Patients diagnosed with schizophrenia seemed to show a higher degree of differences when compared to the other groups of mental disorders, however, the differences were not found to be statistically significant as the p‐values were just above the level of significance (*p* > 0.001), except for the WLM26 parameter between schizophrenia and psychosis comparison that indicated a significant difference (*p* = 0.001). The WLM26 (*p* = 0.0012) and WLM27 (*p* = 0.0015) measurement parameters were just above the significance level for the comparison between the schizophrenic and bipolar mental disorders. The WLM26 (*p* = 0.001), WLM27 (*p* = 0.002) and WLM28 (*p* = 0.0097) measurement parameters were just above the significance level for the comparison between the schizophrenic and psychosis mental disorders.

### 3.4 Shape differences

**FIGURE 2:**
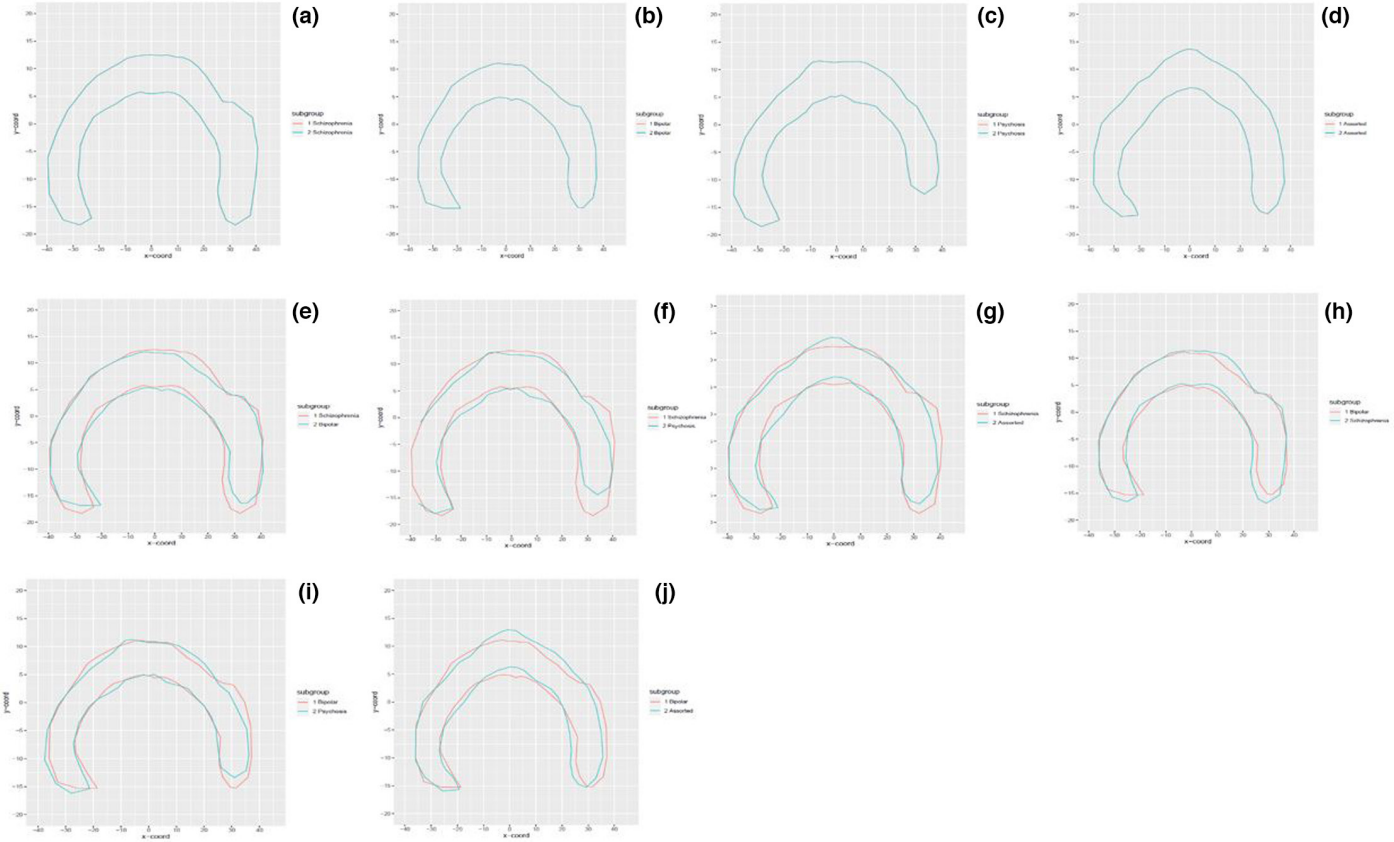
An Ordinary Procrustes analysis between the different mental disorders in the CT sample. (a) Schizophrenia CC shape. (b) Bipolar CC shape. (c) Psychosis CC shape. (d) Various other mental disorders CC shape. (e) Schizophrenia versus Bipolar CC shape. (f) Schizophrenia versus Psychosis CC shape. (g) Schizophrenia versus other various mental disorders CC shape. (h) Bipolar versus Schizophrenia CC shape. (i) Bipolar versus Psychosis CC shape. (j) Bipolar versus other various mental disorders CC shape.

An OPA was performed to obtain plots for comparisons of the CC shape between the different mental disorders in the CT sample. The Euclidean distance was then calculated to determine the distance between the two CC shapes being compared. The OPA between the different mental disorders in the CT sample is given in Figure 2.

The Euclidean distances as well as the CC shape visualisations from the OPA indicated that the schizophrenic sample had the largest degree of shape variation when compared to the remaining mental disorders. The schizophrenia versus the bipolar sample OPA shapes differed mostly in the genu‐rostrum, posterior body, isthmus and splenium regions of the CC. The schizophrenia versus the psychosis sample shapes differed mostly in the anterior body, posterior body and splenium regions of the CC. The schizophrenia versus the other mental disorders sample shapes differed mostly in the anterior body, mid‐body and isthmus regions of the CC. The bipolar versus the schizophrenia sample shapes differed mostly in the genu‐rostrum, posterior body and splenium regions of the CC. The bipolar versus the psychosis sample shapes differed mostly in the genu‐rostrum, anterior body and splenium regions of the CC. The bipolar versus the other mental disorders sample shapes differed mostly in the anterior body, mid‐body, posterior body, isthmus and splenium regions of the CC (refer to A5).

The RMS of the OPA shape differences indicate that there were differences between all the mental disorders in the CT sample group. With the use of the schizophrenia sample group as the reference shape, the schizophrenia sample group CC shape differed the least with the bipolar sample group followed by the psychosis sample group. The other mental disorders sample group differed the most from the schizophrenia sample group CC shape. With the bipolar sample group as the reference shape, the bipolar sample group CC shape differed the least with the schizophrenia sample group followed by the psychosis sample group. The other mental disorders sample group differed the most from the bipolar sample group CC shape.

### 3.5 Results between CT versus MRI versus cadaver sample groups

Euclidean distance differences in the anatomical regions were observed between the different sample groups. The Euclidean distances as well as the CC shape visualisations from the OPA indicated that the MRI group had the largest degree of overall CC shape variation when compared to the CT and the cadaver group, respectively. The MRI group shape differed the most from the CT group in all the anatomical regions of the CC especially in the splenium, genu‐rostrum and isthmus of the CC, respectively. The cadaver group shape also differed from the CT group in all the anatomical regions of the CC, however, it did not differ as largely as with the MRI group. The anatomical regions where the cadaver group differed the most from the CT group were at the isthmus and the mid‐body regions of the CC, respectively. The observations indicated that the CC shape of the CT and the cadaver sample groups were more similar than compared to the MRI sample group CC shape (refer to A6–A8).

The RMS of the OPA shape differences indicate that there were differences between all the groups as well as the overall sample sex. With the use of the CT sample group as the reference shape, the CT sample group shape differed the least with the cadaver sample group followed by the MRI sample group. With the cadaver sample group as the reference shape, the cadaver group CC shape differed the least with the CT sample group and differed the most with the MRI group CC shape. Hotelling's T‐squared test results indicated statistically significant (*p* < 0.05) differences between the MRI and CT samples (*p* = 0.00092) and the MRI and cadaver samples (*p* = 0.003556) only in the isthmus region of the CC.

Differences between the overall CC shape, and the different anatomical regions of the CC, using the MRI sample group CC shape as the reference shape that was compared to the schizophrenic and bipolar mental disorder CC shapes indicated that the schizophrenic mental disorder CC shape differed the most from the MRI reference CC shape. The schizophrenia CC shape differed in all the anatomical regions of the CC, with the most difference observed in the splenium, genu‐rostrum, isthmus, posterior body, mid‐body and anterior body, respectively. The bipolar CC shape also differed from the MRI reference CC shape, however, not to the degrees of difference observed with the schizophrenic CC shape. The bipolar CC shape differed in all the anatomical regions of the CC, with the most difference observed in the posterior body, splenium, genu‐rostrum, isthmus, mid‐body and anterior body, respectively (refer to A9).

In the present study, the level of intra‐ and inter‐rater agreement was found to be between 0.81–1.00, therefore, indicating that the present study appropriately accounted for accuracy and reliability of the methodology and the results obtained.

## 4 DISCUSSION

The present study measured CC shape by use of quantitative morphological data of the CC and combining the data with existing known shape descriptors to analyse the CC shape. A radial partitioning approach by (Clarke et al., 1989) was used in combination with landmarks, described by (Downhill et al., 2000), that correspond to the anatomical subsections of the CC accounting for increased measurement accuracy and sensitivity of any shape differences. The CC was analysed further in relation to its six subregions which were defined from a combination of the (Witelson, 1989) subregions and the (Hampel et al., 1998) subregions that appropriately fit into the radial partitioning approach and the plotted landmarks. This methodological approach was used in order to elaborate on anatomical shape differences of the global CC, as it was essential to gain a deeper understanding on the functional specificity of the CC.

### 4.1 Corpus callosum global shape differences between different sample groups

Research studies that have focussed on the anatomy of the CC have predominantly used one specific scan modality, such as MRI scans, in order to conduct morphological analysis on the CC (Gupta et al., 2008a, 2008b). There have been very few studies that have used different sample modalities to investigate CC morphology as well as to compare the data between the different modalities (Gupta et al., 2008a, 2008b). The present study used cadaver brain images, MRI and CT scans to conduct morphological analysis on the CC, providing a unique and holistic approach to reporting the shape differences observed in the CC.

In the present study, statistically significant CC shape and size differences were found between the three sample groups for numerous CC measurement parameters defining the global shape of the CC. Comparison between the control groups (MRI and cadaver) did not show a high degree of significant differences in the global CC shape parameters, however, the MRI and cadaver control groups differed significantly from the mental disorder CT group. This provides evidence that the CC undergoes global morphological changes that result from various mental disorders.

The MRI group had the largest degree of overall CC shape variation when compared to the CT and the cadaver group, respectively. These observations indicated that the CC shape of the CT and the cadaver sample groups were more similar when compared to the MRI sample group CC shape. This may indicate that the embalming process may have caused minor distortion of the CC shape. It has been suggested that formalin fixation may cause the brain to distort and shrink by more or less 5% (Gupta et al., 2008a, 2008b).

### 4.2 Shape differences in CC anatomical regions between groups and sub‐groups

#### 4.2.1 The genu‐rostrum region

The WLM5 parameter was the only shape parameter to show significant differences in the overall group comparisons (CAD vs. MRI vs. CT). The WLM5 parameter defines the endpoint of the genu‐rostrum and the beginning of the anterior body, indicating that the thickness of the genu‐rostrum at this landmark is thinner in the healthy MRI sample when compared to the mental disorder patient CT and cadaver sample groups. This finding is in contrast to the literature. A possible reason for these results may be due to the disproportionate sample group distribution as the MRI sample consisted of most of the entire study sample.

In contrast to the results of the present study, studies by Walterfang et al. (2008), Downhill et al. (2000) and Huang et al. (2019) reported significant differences in the shape of the genu between healthy controls and schizophrenic patients. These studies further reported that the genu of schizophrenic patients had a smaller area and thickness compared to their healthy counterparts, which was also in contrast to the finding of the present study that indicated larger genu thickness in the schizophrenic sub‐group compared to healthy controls. The decrease in thickness and reduced area in the genu may result in diminished cognitive performance due to fibre integrity changes that cause dysfunction of information transfer between the frontal association cortical regions that are responsible for governing executive functioning (Downhill et al., 2000). In the present study, the schizophrenic population only made up a very small proportion of the CT sample group. A study by Prendergast et al. (2015) suggested that the thickness of the CC, specifically in the genu, reaches peak thickness in males at age 32 and females at age 40, therefore, providing a possible reason for the larger genu seen with schizophrenic patients in the present study.

#### 4.2.2 The body and isthmus regions

It was found that the MRI group differed from both the cadaver and CT groups in the body and isthmus regions of the CC. The WLM19‐20 parameters in the posterior body sub‐region and the WLM21‐22 parameters included in the isthmus region of the CC showed significant differences. These specific shape parameters define the end of the posterior body and the beginning of the isthmus region which is the location of the CC that has been implicated in undergoing shape changes in specific mental disorders such as schizophrenia and bipolar disorder.

The CC shape parameter results indicated that the MRI group's body and isthmus region was thinner when compared to the cadaver and CT groups. These findings are in contrast to Prendergast et al. (2018). Another study by Walterfang et al. (2008) reported regional thickness reductions in the body and isthmus regions of the CC of schizophrenic and first‐episode schizophrenic groups when compared to healthy controls that may be associated with the progression of the mental disorder and or reductions in the fibre number in the region due to increasing age. Therefore, the findings of the present study may be as a result of comparing different scan modalities with different resolutions and not accounting for equal distribution of matched age and sex comparisons between the study sample groups.

OPA shape analysis of the body and isthmus regions between the three sample groups indicated no significant shape differences (*p* > 0.05) present in the body region of the CC. The isthmus region presented significant shape differences between the MRI and cadaver group (*p* = 0.003) as well as between the MRI and CT group (*p* = 0.0009). The results further indicated that the healthy MRI control group differed significantly from the schizophrenic CT sub‐group in the anterior body sub‐region (*p* = 0.027) and isthmus region (*p* = 0.001). A significant difference was also observed between the healthy MRI control group and the bipolar CT sub‐group in the isthmus region of the CC (*p* = 0.009). These results are in agreement with the results reported in Downhill et al. (2000) and Walterfang et al. (2008). This indicates significant negative correlations with cognitive performance (Downhill et al., 2000). Studies have suggested that the reductions in size and shape changes of the anterior body and isthmus may be due to a decrease in the number of axonal fibres connecting cortical areas. The anterior body/posterior genu and isthmus regions connect the prefrontal, temporal and inferior parietal cortical regions (Walterfang et al., 2008). These cortical regions have been found to be affected in schizophrenic patients.

Therefore, in the present study, it was established that significant shape differences were present in the isthmus between the MRI and cadaver group as well as for the MRI and CT group. Statistical significance in the anterior body sub‐region and the isthmus region was found between the healthy MRI control group and the schizophrenia CT sup‐group. Significant differences between the healthy MRI control group and the bipolar CT sub‐group were observed in the isthmus region of the CC.

#### 4.2.3 The splenium region

The MRI group significantly differed with the CT group in all the splenium shape parameters, however, the MRI group did not significantly differ with the cadaver group for any of the splenium shape parameters. The CT group significantly differed with the cadaver group in all the splenium shape parameters. The CC shape parameter results indicated that the MRI and cadaver group's splenium regions were thinner when compared to the CT group. Francis et al. (2016) and Prendergast et al. (2018) found that the posterior splenium region in schizophrenic and bipolar patients were smaller in volume compared to healthy controls. The smaller splenium regions may indicate dysfunction of this region causing interhemispheric communication abnormalities between the visual cortices and executive functioning pre‐frontal and temporo‐parietal association cortices that result in impairment of cognitive function and symptoms presented by schizophrenic patients (Downhill et al., 2000; Francis et al., 2016).

In the present study, it was established that significant shape differences were present in the splenium of the CC between the three sample groups for the splenial parameters, however, no statistically significant differences were observed from the OPA shape difference, providing a robust perspective on the differences found. Therefore, the result of the splenium needs to be interpreted with caution, suggesting that no significant differences were observed between healthy controls and mental disorders in this region.

## 5 CONCLUSION

- No significant shape differences were found between the different mental disorders in the CT sample group.
  - Although not significant, schizophrenia and bipolar CC shapes differed mostly in the genu‐rostrum, posterior body, isthmus and splenium.
  - Schizophrenia and psychosis CC shapes differed mostly in the anterior body, posterior body and splenium.
  - Bipolar and psychosis CC shapes differed mostly in the genu‐rostrum, anterior body and splenium.
  - The schizophrenia sample differed the most in the posterior CC when compared to bipolar and psychosis, specifically in the splenium.
- Sample group comparisons yielded significant differences between all sample groups for all global CC measurement parameters.
- Significant shape differences were also observed in the various CC sub‐regions between the cadaver, MRI and CT groups as well as between the MRI, schizophrenic and bipolar groups.
  - The isthmus subregion showed statistical significance between the MRI and CT group comparison as well as the MRI and cadaver group comparison.
  - Statistically significant shape differences were found in the anterior body and isthmus sub‐regions of the CC between the healthy MRI group and the schizophrenia CT sub‐group. Statistically significant shape differences were found in the isthmus subregion of the CC between the healthy MRI group and the bipolar CT sub‐group.
- The findings of the present study suggest that the CC in schizophrenia and bipolar differs significantly compared to healthy controls, specifically in the anterior body and isthmus for schizophrenia and only in the isthmus for bipolar disorder. As these sub‐regions have been shown to interconnect the prefrontal, temporal and inferior parietal cortical regions that govern specific cognitive functions, shape changes in these regions may, in part, be responsible for the symptoms and cognitive impairments observed in schizophrenic and bipolar patients when shape and size changes occur in these regions. Therefore, these sub‐regions may be of interest to the diagnostic process of schizophrenia and bipolar mental disorders.

## AUTHOR CONTRIBUTIONS

All authors contributed to the study conception and design. Material preparation, data collection and analysis were performed by Mr Christiaan L Vermeulen, Dr Gerda Venter and Dr René Human‐Baron. The first draft of the manuscript was written by Mr Christiaan L Vermeulen and all authors commented on previous versions of the manuscript. All authors read and approved the final manuscript.

## FUNDING INFORMATION

The authors did not receive support from any organisation for the submitted work. All authors certify that they have no affiliations with or involvement in any organisation or entity with any financial interest or non‐financial interest in the subject matter or materials discussed in this manuscript.

## CONFLICT OF INTEREST

The authors declare that they have no conflict of interest. The authors have no competing interests to declare that are relevant to the content of this article.

## ETHICS STATEMENT

The study was of a retrospective nature as a database containing digital cerebral photos, MRI scans and CT scans were used, which was already available in the Department of Anatomy at the University of Pretoria. This study was performed in line with the principles of the Declaration of Helsinki. This study was approved by the Faculty of Health Sciences Research Ethics Committee (555/2018). The use of cadaver tissue for research is covered by the National Health Act, 61 of 2003. Permission for the use of the cadaver brain specimens was obtained from the Departments of Anatomy at the University of Pretoria, Sefako Makgatho Health Sciences University and the University of Witwatersrand. Permission for the use of the MRI and CT scans was also granted by the Radiology Department of Mediclinic Muelmed Hospital and Kalafong Provincial Tertiary Hospital, respectively.

## Supporting information


**Appendix S1:** Supporting Information

## Data Availability

All data has been added as supplementary materials.
