# Supplementary material for: A morphological study of the shape of the corpus callosum in normal, schizophrenic and bipolar patients
Source: J Anat. 2022 Oct 13;242(2):153–63. doi: 10.1111/joa.13777 (PMC9877476; doi:10.1111/joa.13777)
Supplement: Supplementary file 1 — Appendix S1: Supporting Information [file JOA-242-153-s001.docx]

SUPPLEMENTARY MATERIAL

A1: Descriptive statistics of CC shape parameters of the cadaver sample (n=50)

| **Measurement Parameter** | **Min (mm)** | **Max (mm)** | **Mean (mm)** | **SD (mm)** | **95% CI Lower** | **95% CI Upper** |
| --- | --- | --- | --- | --- | --- | --- |
| OT Area | 426.33 | 862.52 | 644.37 | 98.52 | 617 | 672 |
| OT Roundness | 0.25 | 0.39 | 0.31 | 0.04 | 0.303 | 0.322 |
| TH | 20.89 | 32.16 | 26.45 | 2.57 | 25.7 | 27.2 |
| TL | 61.20 | 82.54 | 71.48 | 4.60 | 70.2 | 72.8 |
| GW | 6.33 | 12.21 | 9.09 | 1.18 | 8.75 | 9.42 |
| ABW | 4.22 | 9.45 | 6.35 | 1.26 | 6 | 6.7 |
| PBW | 2.88 | 7.51 | 5.21 | 0.96 | 4.94 | 5.48 |
| ISW | 6.49 | 12.45 | 9.32 | 1.40 | 8.93 | 9.71 |
| SC Area | 769.53 | 1520.73 | 1085.49 | 189.11 | 1033 | 1138 |
| SC Roundness | 0.30 | 0.50 | 0.39 | 0.05 | 0.376 | 0.405 |
| WLM1 | 3.78 | 7.48 | 5.56 | 0.99 | 5.29 | 5.84 |
| WLM2 | 6.31 | 12.40 | 9.71 | 1.47 | 9.3 | 10.1 |
| WLM3 | 8.45 | 15.50 | 12.06 | 1.58 | 11.6 | 12.5 |
| WLM4 | 8.44 | 14.02 | 11.40 | 1.41 | 11 | 11.8 |
| WLM5 | 6.35 | 12.40 | 9.09 | 1.22 | 8.75 | 9.43 |
| WLM6 | 4.73 | 9.71 | 7.42 | 1.14 | 7.1 | 7.74 |
| WLM7 | 3.60 | 8.42 | 6.55 | 1.24 | 6.21 | 6.89 |
| WLM8 | 3.81 | 8.19 | 6.18 | 1.19 | 5.85 | 6.51 |
| WLM9 | 3.23 | 8.89 | 6.16 | 1.31 | 5.79 | 6.52 |
| WLM10 | 4.13 | 9.45 | 6.44 | 1.27 | 6.09 | 6.79 |
| WLM11 | 4.33 | 9.29 | 6.54 | 1.23 | 6.2 | 6.88 |
| WLM12 | 4.39 | 9.26 | 6.56 | 1.08 | 6.26 | 6.87 |
| WLM13 | 4.88 | 8.40 | 6.62 | 0.94 | 6.35 | 6.89 |
| WLM14 | 4.25 | 8.99 | 6.77 | 0.98 | 6.5 | 7.04 |
| WLM15 | 4.49 | 8.77 | 6.79 | 0.95 | 6.52 | 7.05 |
| WLM16 | 3.59 | 8.43 | 6.38 | 1.10 | 6.08 | 6.69 |
| WLM17 | 3.71 | 8.14 | 6.09 | 1.02 | 5.81 | 6.37 |
| WLM18 | 3.90 | 7.67 | 5.85 | 0.88 | 5.6 | 6.1 |
| WLM19 | 2.91 | 7.30 | 5.43 | 1.01 | 5.14 | 5.71 |
| WLM20 | 2.71 | 7.29 | 5.29 | 0.98 | 5.01 | 5.56 |
| WLM21 | 3.46 | 7.62 | 5.56 | 1.02 | 5.28 | 5.84 |
| WLM22 | 3.93 | 8.92 | 6.15 | 1.20 | 5.81 | 6.49 |
| WLM23 | 2.99 | 9.91 | 6.80 | 1.52 | 6.38 | 7.22 |
| WLM24 | 4.42 | 11.88 | 7.88 | 1.57 | 7.45 | 8.32 |
| WLM25 | 5.35 | 12.39 | 9.32 | 1.51 | 8.9 | 9.74 |
| WLM26 | 6.63 | 14.35 | 10.96 | 1.72 | 10.5 | 11.4 |
| WLM27 | 8.45 | 16.60 | 11.81 | 1.86 | 11.3 | 12.3 |
| WLM28 | 6.69 | 12.91 | 10.59 | 1.36 | 10.2 | 11 |
| WLM29 | 5.09 | 8.41 | 6.49 | 0.82 | 6.26 | 6.72 |
| APC | 22.14 | 33.54 | 27.30 | 2.67 | 26.5 | 28.1 |
| ALM-APC | 19.56 | 27.13 | 24.12 | 2.06 | 23.5 | 24.7 |
| ILM5-APC | 27.87 | 37.59 | 32.68 | 2.21 | 32.1 | 33.3 |
| ILM10-APC | 21.04 | 35.42 | 27.50 | 3.46 | 26.5 | 28.5 |
| ILM15-APC | 16.70 | 33.35 | 25.41 | 3.77 | 24.4 | 26.5 |
| ILM20-APC | 16.51 | 31.00 | 24.21 | 3.50 | 23.2 | 25.2 |
| ILM25-APC | 16.43 | 31.57 | 24.14 | 3.08 | 23.3 | 25 |
| PLM-APC | 18.39 | 30.37 | 24.98 | 2.66 | 24.2 | 25.7 |
| SLM5-APC | 35.24 | 47.22 | 40.49 | 2.60 | 39.8 | 41.2 |
| SLM10-APC | 27.11 | 40.80 | 33.72 | 3.73 | 32.7 | 34.8 |
| SLM15-APC | 23.76 | 40.80 | 31.92 | 3.77 | 30.9 | 33 |
| SLM20-APC | 21.13 | 36.91 | 29.50 | 3.76 | 28.5 | 30.5 |
| SLM25-APC | 26.68 | 39.01 | 32.61 | 2.93 | 31.8 | 33.4 |
| ANGLE | 84.79 | 118.27 | 100.17 | 8.42 | 97.8 | 103 |
| AGE | 26 | 95 | 69.27 | 18.24 | 63.9 | 74.7 |

A2: Descriptive statistics of CC shape parameters of the MRI sample (n=111)

| **Measurement Parameter** | **Min (mm)** | **Max (mm)** | **Mean (mm)** | **SD (mm)** | **95% CI Lower** | **95% CI Upper** |
| --- | --- | --- | --- | --- | --- | --- |
| OT Area | 418.57 | 854.63 | 611.01 | 94.37 | 593 | 629 |
| OT Roundness | 0.20 | 0.39 | 0.28 | 0.04 | 0.27 | 0.29 |
| TH | 18.06 | 32.64 | 25.08 | 3.08 | 24.5 | 25.7 |
| TL | 60.84 | 89.62 | 75.35 | 5.58 | 74.3 | 76.4 |
| GW | 4.95 | 11.07 | 8.15 | 1.33 | 7.9 | 8.4 |
| ABW | 3.14 | 7.46 | 5.44 | 0.88 | 5.27 | 5.6 |
| PBW | 2.52 | 6.46 | 4.35 | 0.93 | 4.17 | 4.52 |
| ISW | 6.36 | 12.44 | 9.18 | 1.28 | 8.94 | 9.43 |
| SC Area | 709.45 | 1505.55 | 1095.69 | 180.99 | 1061 | 1130 |
| SC Roundness | 0.24 | 0.46 | 0.35 | 0.05 | 0.337 | 0.355 |
| WLM1 | 2.61 | 8.11 | 5.33 | 1.05 | 5.13 | 5.52 |
| WLM2 | 5.29 | 14.18 | 9.57 | 1.62 | 9.26 | 9.87 |
| WLM3 | 8.45 | 16.18 | 12.08 | 1.69 | 11.8 | 12.4 |
| WLM4 | 7.49 | 14.69 | 10.90 | 1.52 | 10.6 | 11.2 |
| WLM5 | 4.55 | 11.53 | 8.17 | 1.40 | 7.91 | 8.43 |
| WLM6 | 3.28 | 9.95 | 6.35 | 1.17 | 6.13 | 6.57 |
| WLM7 | 3.02 | 8.63 | 5.74 | 1.08 | 5.54 | 5.94 |
| WLM8 | 3.41 | 8.26 | 5.51 | 1.02 | 5.31 | 5.7 |
| WLM9 | 3.14 | 7.42 | 5.45 | 0.97 | 5.27 | 5.63 |
| WLM10 | 3.39 | 7.24 | 5.52 | 0.88 | 5.35 | 5.68 |
| WLM11 | 3.18 | 8.08 | 5.61 | 0.91 | 5.44 | 5.78 |
| WLM12 | 3.36 | 7.92 | 5.67 | 0.89 | 5.51 | 5.84 |
| WLM13 | 3.66 | 7.74 | 5.68 | 0.87 | 5.52 | 5.85 |
| WLM14 | 4.07 | 7.57 | 5.78 | 0.84 | 5.62 | 5.94 |
| WLM15 | 3.92 | 7.87 | 5.85 | 0.86 | 5.69 | 6.01 |
| WLM16 | 3.67 | 7.78 | 5.69 | 0.86 | 5.53 | 5.85 |
| WLM17 | 3.28 | 7.27 | 5.40 | 0.82 | 5.25 | 5.56 |
| WLM18 | 3.14 | 6.87 | 5.00 | 0.74 | 4.86 | 5.14 |
| WLM19 | 2.75 | 6.43 | 4.59 | 0.78 | 4.44 | 4.73 |
| WLM20 | 2.49 | 6.58 | 4.44 | 0.92 | 4.26 | 4.61 |
| WLM21 | 2.71 | 7.06 | 4.52 | 1.00 | 4.33 | 4.71 |
| WLM22 | 2.78 | 8.78 | 5.27 | 1.26 | 5.04 | 5.51 |
| WLM23 | 3.14 | 9.75 | 6.34 | 1.30 | 6.1 | 6.59 |
| WLM24 | 4.22 | 10.44 | 7.59 | 1.28 | 7.35 | 7.83 |
| WLM25 | 6.06 | 12.14 | 9.26 | 1.25 | 9.02 | 9.5 |
| WLM26 | 7.56 | 15.45 | 11.16 | 1.59 | 10.9 | 11.5 |
| WLM27 | 7.72 | 15.74 | 11.96 | 1.67 | 11.6 | 12.3 |
| WLM28 | 6.48 | 14.31 | 10.59 | 1.61 | 10.3 | 10.9 |
| WLM29 | 4.45 | 8.50 | 6.39 | 0.84 | 6.22 | 6.55 |
| APC | 23.15 | 31.24 | 26.70 | 1.69 | 26.4 | 27 |
| ALM-APC | 19.52 | 33.30 | 26.47 | 2.58 | 26 | 27 |
| ILM5-APC | 27.72 | 39.80 | 34.00 | 2.56 | 33.5 | 34.5 |
| ILM10-APC | 21.30 | 33.02 | 27.34 | 2.60 | 26.8 | 27.8 |
| ILM15-APC | 17.32 | 30.41 | 24.29 | 2.65 | 23.8 | 24.8 |
| ILM20-APC | 18.01 | 30.39 | 23.80 | 2.68 | 23.3 | 24.3 |
| ILM25-APC | 18.45 | 33.07 | 25.55 | 3.15 | 25 | 26.1 |
| PLM-APC | 19.73 | 34.41 | 26.95 | 3.07 | 26.4 | 27.5 |
| SLM5-APC | 34.97 | 47.69 | 41.05 | 2.70 | 40.5 | 41.6 |
| SLM10-APC | 26.09 | 40.23 | 32.75 | 2.67 | 32.2 | 33.3 |
| SLM15-APC | 23.96 | 36.92 | 30.09 | 2.66 | 29.6 | 30.6 |
| SLM20-APC | 22.17 | 35.34 | 28.29 | 2.75 | 27.8 | 28.8 |
| SLM25-APC | 26.68 | 40.72 | 33.65 | 3.22 | 33 | 34.3 |
| ANGLE | 83.26 | 130.09 | 107.20 | 9.71 | 105 | 109 |
| AGE | 20 | 83 | 46.10 | 15.64 | 43.2 | 49 |

A3: Descriptive statistics of CC shape parameters of the CT sample (n=62)

| **Measurement Parameter** | **Min (mm)** | **Max (mm)** | **Mean (mm)** | **SD (mm)** | **95% CI Lower** | **95% CI Upper** |
| --- | --- | --- | --- | --- | --- | --- |
| OT Area | 466.23 | 1073.78 | 769.50 | 134.52 | 735 | 804 |
| OT Roundness | 0.20 | 0.40 | 0.31 | 0.04 | 0.3 | 0.32 |
| TH | 21.10 | 38.43 | 29.00 | 3.92 | 28 | 30 |
| TL | 63.99 | 91.39 | 78.48 | 6.03 | 76.9 | 80 |
| GW | 5.41 | 13.67 | 9.42 | 1.72 | 8.99 | 9.85 |
| ABW | 3.37 | 9.21 | 6.71 | 1.14 | 6.43 | 7 |
| PBW | 3.42 | 9.60 | 6.20 | 1.31 | 5.87 | 6.53 |
| ISW | 5.62 | 13.45 | 9.65 | 1.85 | 9.18 | 10.1 |
| SC Area | 828.90 | 2024.13 | 1361.83 | 308.32 | 1284 | 1440 |
| SC Roundness | 0.25 | 0.50 | 0.39 | 0.06 | 0.38 | 0.40 |
| WLM1 | 3.05 | 7.90 | 5.45 | 1.21 | 5.14 | 5.75 |
| WLM2 | 2.99 | 15.14 | 9.53 | 2.51 | 8.91 | 10.2 |
| WLM3 | 3.76 | 17.82 | 11.88 | 2.98 | 11.1 | 12.6 |
| WLM4 | 6.49 | 17.05 | 11.53 | 2.35 | 10.9 | 12.1 |
| WLM5 | 5.43 | 13.59 | 9.44 | 1.73 | 9.01 | 9.87 |
| WLM6 | 4.58 | 10.42 | 7.38 | 1.28 | 7.06 | 7.7 |
| WLM7 | 4.57 | 9.44 | 6.87 | 1.08 | 6.6 | 7.15 |
| WLM8 | 3.89 | 9.55 | 6.77 | 1.15 | 6.49 | 7.06 |
| WLM9 | 3.67 | 9.45 | 6.72 | 1.12 | 6.44 | 7 |
| WLM10 | 4.88 | 9.11 | 6.86 | 1.06 | 6.59 | 7.13 |
| WLM11 | 4.82 | 9.30 | 6.90 | 1.07 | 6.63 | 7.17 |
| WLM12 | 4.70 | 9.19 | 6.81 | 1.01 | 6.55 | 7.06 |
| WLM13 | 5.07 | 9.10 | 6.83 | 1.04 | 6.56 | 7.09 |
| WLM14 | 4.99 | 9.19 | 6.80 | 1.07 | 6.53 | 7.07 |
| WLM15 | 3.63 | 9.34 | 6.79 | 1.17 | 6.49 | 7.08 |
| WLM16 | 4.05 | 9.23 | 6.74 | 1.14 | 6.45 | 7.02 |
| WLM17 | 4.28 | 8.94 | 6.62 | 1.06 | 6.35 | 6.89 |
| WLM18 | 4.54 | 9.14 | 6.48 | 1.16 | 6.19 | 6.77 |
| WLM19 | 3.75 | 8.99 | 6.32 | 1.21 | 6.01 | 6.62 |
| WLM20 | 3.45 | 9.71 | 6.28 | 1.32 | 5.94 | 6.61 |
| WLM21 | 3.77 | 8.70 | 6.32 | 1.18 | 6.02 | 6.62 |
| WLM22 | 4.92 | 9.78 | 7.00 | 1.14 | 6.7 | 7.29 |
| WLM23 | 3.98 | 11.79 | 7.48 | 1.52 | 7.09 | 7.87 |
| WLM24 | 4.65 | 12.82 | 8.28 | 1.87 | 7.81 | 8.76 |
| WLM25 | 5.61 | 16.11 | 9.87 | 2.08 | 9.35 | 10.4 |
| WLM26 | 6.72 | 19.79 | 12.53 | 2.71 | 11.9 | 13.2 |
| WLM27 | 7.67 | 19.56 | 13.38 | 2.52 | 12.7 | 14 |
| WLM28 | 7.81 | 16.91 | 11.85 | 2.08 | 11.3 | 12.4 |
| WLM29 | 4.18 | 10.33 | 7.09 | 1.38 | 6.73 | 7.44 |
| APC | 25.04 | 46.77 | 34.02 | 4.66 | 32.9 | 35.2 |
| ALM-APC | 19.13 | 33.09 | 26.01 | 3.48 | 25.1 | 26.9 |
| ILM5-APC | 28.42 | 43.17 | 35.69 | 3.58 | 34.8 | 36.6 |
| ILM10-APC | 20.91 | 40.42 | 30.81 | 4.11 | 29.8 | 31.8 |
| ILM15-APC | 19.93 | 39.30 | 28.43 | 4.27 | 27.4 | 29.5 |
| ILM20-APC | 16.55 | 40.66 | 27.45 | 4.49 | 26.3 | 28.6 |
| ILM25-APC | 21.44 | 36.99 | 27.67 | 3.66 | 26.7 | 28.6 |
| PLM-APC | 21.96 | 37.48 | 30.01 | 4.04 | 29 | 31 |
| SLM5-APC | 35.86 | 53.55 | 44.29 | 4.12 | 43.3 | 45.3 |
| SLM10-APC | 26.35 | 48.74 | 37.77 | 4.55 | 36.6 | 38.9 |
| SLM15-APC | 25.79 | 44.96 | 35.13 | 4.11 | 34.1 | 36.2 |
| SLM20-APC | 21.38 | 42.92 | 33.49 | 4.48 | 32.4 | 34.6 |
| SLM25-APC | 27.69 | 48.46 | 37.19 | 4.43 | 36.1 | 38.3 |
| ANGLE | 78.10 | 127.82 | 102.10 | 10.49 | 99.5 | 105 |
| AGE | 18 | 71 | 38.68 | 15.06 | 34.8 | 42.5 |

A4: Mann-Whitney U bivariate comparison between mental disorders within the CT sample group

| **Measurement Parameters** | **Other**  **vs**  **Bipolar** | **Other**  **vs Psychosis** | **Other**  **vs Schizophrenia** | **Bipolar**  **vs Psychosis** | **Bipolar**  **vs Schizophrenia** | **Psychosis**  **vs Schizophrenia** | |
| --- | --- | --- | --- | --- | --- | --- | --- |
| **Sig. (p<0.001)** | | | | | | |  |
| OT Area | 0.03924 | 0.26419 | 0.41502 | 0.50545 | 0.01911 | 0.11713 | |
| OT Roundness | 0.75527 | 0.30547 | 0.37501 | 0.2099 | 0.34452 | 0.68074 | |
| TH | 0.07606 | 0.25863 | 0.92361 | 0.61058 | 0.11241 | 0.38879 | |
| TL | 0.0615 | 0.65517 | 0.29151 | 0.04574 | 0.05878 | 0.61058 | |
| GW | 0.01761 | 0.43063 | 0.61461 | 0.04574 | 0.01261 | 0.30844 | |
| ABW | 0.03489 | 0.63631 | 0.09805 | 0.11713 | 0.49629 | 0.38879 | |
| PBW | 0.92361 | 0.97904 | 0.92361 | 0.87549 | 1 | 0.90645 | |
| ISW | 0.08434 | 0.33105 | 0.3376 | 0.30844 | 0.01261 | 0.0656 | |
| SC Area | 0.09332 | 0.41543 | 0.96176 | 0.3676 | 0.17362 | 0.61058 | |
| SC Roundness | 0.84789 | 0.2268 | 0.61461 | 0.38875 | 0.87978 | 0.33707 | |
| WLM1 | 0.443 | 0.35785 | 0.41502 | 0.90645 | 0.32575 | 0.1366 | |
| WLM2 | 0.7013 | 0.64569 | 0.27013 | 0.72442 | 0.19876 | 0.22461 | |
| WLM3 | 0.38812 | 0.9581 | 0.3376 | 0.38879 | 0.11241 | 0.43336 | |
| WLM4 | 0.09328 | 0.73271 | 0.565 | 0.17036 | 0.0821 | 0.43336 | |
| WLM5 | 0.02423 | 0.44616 | 0.7013 | 0.07794 | 0.01911 | 0.23992 | |
| WLM6 | 0.13719 | 0.10905 | 0.17173 | 0.81418 | 0.93974 | 0.84472 | |
| WLM7 | 0.27013 | 0.14125 | 0.03924 | 0.81418 | 0.28992 | 0.53081 | |
| WLM8 | 0.17943 | 0.19345 | 0.00628 | 0.41072 | 0.17362 | 0.21001 | |
| WLM9 | 0.17943 | 0.31815 | 0.08434 | 0.21001 | 0.93974 | 0.22461 | |
| WLM10 | 0.03924 | 0.58117 | 0.13719 | 0.12658 | 0.5967 | 0.4567 | |
| WLM11 | 0.05513 | 0.51134 | 0.15032 | 0.22461 | 0.70546 | 0.50545 | |
| WLM12 | 0.03096 | 0.61768 | 0.443 | 0.0921 | 0.13057 | 0.66654 | |
| WLM13 | 0.04932 | 0.38599 | 0.36231 | 0.30844 | 0.25684 | 0.84472 | |
| WLM14 | 0.09332 | 0.61768 | 0.7013 | 0.3676 | 0.32575 | 0.96875 | |
| WLM15 | 0.08434 | 0.52838 | 0.7013 | 0.33715 | 0.19876 | 0.90645 | |
| WLM16 | 0.23066 | 0.5633 | 0.59791 | 0.81418 | 0.28992 | 0.96875 | |
| WLM17 | 0.63161 | 0.51134 | 0.84791 | 0.86006 | 0.44969 | 0.87549 | |
| WLM18 | 0.88563 | 0.8131 | 0.84791 | 0.96875 | 0.70546 | 0.75399 | |
| WLM19 | 0.96176 | 0.8541 | 0.81054 | 0.96875 | 0.76237 | 0.66654 | |
| WLM20 | 0.84791 | 0.91631 | 0.84791 | 1 | 0.87983 | 0.78392 | |
| WLM21 | 0.47202 | 0.63631 | 0.96176 | 0.87549 | 0.70546 | 0.5568 | |
| WLM22 | 0.06846 | 0.37175 | 0.7013 | 0.29021 | 0.02837 | 0.21001 | |
| WLM23 | 0.03489 | 0.18901 | 0.56505 | 0.07794 | 0.02334 | 0.14723 | |
| WLM24 | 0.04932 | 0.43063 | 0.75527 | 0.07794 | 0.05878 | 0.25595 | |
| WLM25 | 0.07606 | 0.33105 | 0.38812 | 0.34714 | 0.03429 | 0.07155 | |
| WLM26 | 0.38812 | 0.34429 | 0.00252 | 0.96875 | 0.00115 | 0.001 | |
| WLM27 | 0.53309 | 0.41543 | 0.00155 | 0.81418 | 0.0015 | 0.00197 | |
| WLM28 | 0.96176 | 0.43063 | 0.00628 | 0.90645 | 0.01261 | 0.00973 | |
| WLM29 | 0.84791 | 0.61768 | 0.00542 | 0.87549 | 0.02837 | 0.01875 | |
| APC | 0.23066 | 0.87475 | 0.59791 | 0.08478 | 0.36435 | 0.53081 | |
| ALM-APC | 0.50206 | 0.02228 | 0.21254 | 0.00973 | 0.11241 | 0.50545 | |
| ILM5-APC | 0.38812 | 0.5993 | 0.47202 | 0.19611 | 0.15093 | 0.93755 | |
| ILM10-APC | 0.81054 | 0.69354 | 0.21254 | 0.90645 | 0.25684 | 0.27271 | |
| ILM15-APC | 0.66609 | 0.49459 | 0.19548 | 0.87549 | 0.19876 | 0.32742 | |
| ILM20-APC | 0.56505 | 0.8541 | 0.24985 | 0.61058 | 0.11241 | 0.23992 | |
| ILM25-APC | 0.53309 | 0.67425 | 0.59791 | 0.48074 | 0.40568 | 0.87549 | |
| PLM-APC | 0.07606 | 0.63631 | 0.59791 | 0.08478 | 0.40568 | 0.27271 | |
| SLM5-APC | 0.1136 | 0.5457 | 0.36231 | 0.04165 | 0.04937 | 0.6383 | |
| SLM10-APC | 0.7013 | 0.61768 | 0.41502 | 0.4567 | 0.40568 | 0.5568 | |
| SLM15-APC | 0.59791 | 0.79278 | 0.314 | 0.43336 | 0.19876 | 0.25595 | |
| SLM20-APC | 0.53309 | 0.91631 | 0.314 | 0.69526 | 0.04937 | 0.21001 | |
| SLM25-APC | 0.10306 | 1 | 0.3376 | 0.25595 | 0.06964 | 0.39963 | |
| ANGLE | 0.56505 | 0.14849 | 0.66609 | 0.07794 | 0.8206 | 0.23992 | |
| AGE | 0.20876 | 0.0199 | 0.01306 | 0.091 | 0.10202 | 0.91312 | |

*Statistically significant.

A5: The overall OPA shape differences between the different mental disorders

| **CT – Mental Disorders** | | | | | |
| --- | --- | --- | --- | --- | --- |
|  | **Psychosis** | **Other Mental Disorders** | **Bipolar** | **Schizophrenia** |  |
| **Psychosis** | 0 | 1.716 | 1.536 | 1.607 |  |
| **Other Mental Disorders** | 1.761 | 0 | 1.808 | 1.785 |  |
| **Bipolar** | 1.455 | 1.716 | 0 | 1.45 |  |
| **Schizophrenia** | 1.721 | 1.852 | 1.519 | 0 |  |

A6: Descriptive statistics of CC shape parameters across all sample groups (n=223)

| **Measurement Parameter** | **N** | **Min (mm)** | **Max (mm)** | **Mean (mm)** | **SD (mm)** | **95% CI Lower** | **95% CI Upper** |
| --- | --- | --- | --- | --- | --- | --- | --- |
| OT Area | 223 | 419 | 958 | 654 | 116 | 638 | 669 |
| OT Roundness | 223 | 0.20 | 0.40 | 0.29 | 0.04 | 0.289 | 0.3 |
| TH | 223 | 17.3 | 35.9 | 26.4 | 3.58 | 25.9 | 26.9 |
| TL | 223 | 58.8 | 91.4 | 75.3 | 6.1 | 74.4 | 76.1 |
| GW | 223 | 4.35 | 12.9 | 8.62 | 1.54 | 8.42 | 8.83 |
| ABW | 223 | 3.13 | 8.82 | 5.95 | 1.14 | 5.8 | 6.1 |
| PBW | 223 | 2.52 | 8.47 | 5.02 | 1.26 | 4.85 | 5.18 |
| ISW | 223 | 5.62 | 13.3 | 9.34 | 1.48 | 9.14 | 9.54 |
| SC Area | 223 | 709 | 1788 | 1144 | 215 | 1114 | 1173 |
| SC Roundness | 223 | 0.24 | 0.50 | 0.37 | 0.06 | 0.36 | 0.38 |
| WLM1 | 223 | 2.6 | 8.11 | 5.41 | 1.08 | 5.27 | 5.56 |
| WLM2 | 223 | 5.29 | 14.2 | 9.6 | 1.83 | 9.35 | 9.84 |
| WLM3 | 223 | 7.04 | 16.8 | 12 | 2.03 | 11.7 | 12.3 |
| WLM4 | 223 | 6.49 | 15.6 | 11.1 | 1.73 | 10.9 | 11.3 |
| WLM5 | 223 | 4.55 | 12.8 | 8.65 | 1.53 | 8.45 | 8.85 |
| WLM6 | 223 | 3.28 | 10.4 | 6.85 | 1.32 | 6.68 | 7.02 |
| WLM7 | 223 | 3.02 | 9.44 | 6.23 | 1.22 | 6.07 | 6.4 |
| WLM8 | 223 | 3.41 | 9.19 | 5.98 | 1.18 | 5.82 | 6.13 |
| WLM9 | 223 | 3.14 | 9.08 | 5.95 | 1.2 | 5.79 | 6.1 |
| WLM10 | 223 | 3.08 | 9.11 | 6.06 | 1.2 | 5.91 | 6.22 |
| WLM11 | 223 | 3.18 | 9.2 | 6.14 | 1.17 | 5.99 | 6.3 |
| WLM12 | 223 | 3.19 | 9.26 | 6.15 | 1.13 | 6 | 6.3 |
| WLM13 | 223 | 3.66 | 9.09 | 6.19 | 1.06 | 6.05 | 6.33 |
| WLM14 | 223 | 4.07 | 8.99 | 6.26 | 1.05 | 6.13 | 6.4 |
| WLM15 | 223 | 3.63 | 8.87 | 6.28 | 1.08 | 6.13 | 6.42 |
| WLM16 | 223 | 3.59 | 8.88 | 6.1 | 1.06 | 5.96 | 6.25 |
| WLM17 | 223 | 3.28 | 8.58 | 5.88 | 1.05 | 5.74 | 6.02 |
| WLM18 | 223 | 2.89 | 8.41 | 5.51 | 1.05 | 5.37 | 5.65 |
| WLM19 | 223 | 2.75 | 8.43 | 5.22 | 1.15 | 5.07 | 5.37 |
| WLM20 | 223 | 2.49 | 8.56 | 5.12 | 1.27 | 4.95 | 5.29 |
| WLM21 | 223 | 2.71 | 9.1 | 5.28 | 1.34 | 5.11 | 5.46 |
| WLM22 | 223 | 2.74 | 9.78 | 5.92 | 1.45 | 5.73 | 6.12 |
| WLM23 | 223 | 2.99 | 10.8 | 6.73 | 1.45 | 6.54 | 6.92 |
| WLM24 | 223 | 4.22 | 11.9 | 7.78 | 1.45 | 7.58 | 7.97 |
| WLM25 | 223 | 5.61 | 13.1 | 9.39 | 1.45 | 9.19 | 9.58 |
| WLM26 | 223 | 6.63 | 15.9 | 11.4 | 1.87 | 11.1 | 11.6 |
| WLM27 | 223 | 7.67 | 17.7 | 12.3 | 2.01 | 12 | 12.5 |
| WLM28 | 223 | 5.87 | 16 | 10.9 | 1.77 | 10.6 | 11.1 |
| WLM29 | 223 | 4.02 | 9.2 | 6.55 | 0.99 | 6.42 | 6.69 |
| APC | 223 | 22.1 | 40 | 28.7 | 3.96 | 28.2 | 29.3 |
| ALM-APC | 223 | 19.1 | 33.1 | 25.8 | 2.9 | 25.4 | 26.2 |
| ILM5-APC | 223 | 27.3 | 40.2 | 33.9 | 2.75 | 33.6 | 34.3 |
| ILM10-APC | 223 | 20.9 | 37.1 | 28.2 | 3.4 | 27.8 | 28.7 |
| ILM15-APC | 223 | 16.4 | 34.8 | 25.6 | 3.69 | 25.1 | 26.1 |
| ILM20-APC | 223 | 16.5 | 33.9 | 24.7 | 3.52 | 24.3 | 25.2 |
| ILM25-APC | 223 | 16.4 | 33.6 | 25.7 | 3.32 | 25.2 | 26.1 |
| PLM-APC | 223 | 18.4 | 37.5 | 27.4 | 3.82 | 26.9 | 27.9 |
| SLM5-APC | 223 | 32.8 | 50.4 | 41.5 | 3.14 | 41.1 | 41.9 |
| SLM10-APC | 223 | 26.1 | 44.6 | 34.3 | 3.89 | 33.8 | 34.8 |
| SLM15-APC | 223 | 21.6 | 41.1 | 31.8 | 3.96 | 31.2 | 32.3 |
| SLM20-APC | 222 | 21.1 | 39.8 | 29.8 | 3.87 | 29.3 | 30.3 |
| SLM25-APC | 223 | 26.7 | 43.3 | 34.2 | 3.61 | 33.7 | 34.7 |
| ANGLE | 223 | 78.1 | 130 | 104 | 10.2 | 103 | 105 |
| AGE | 214 | 18 | 95 | 48.8 | 19.3 | 46.2 | 51.4 |

A7: ANOVA analysis between the groups,

| **Measurement Parameter** | **Group** |  |  |
| --- | --- | --- | --- |
| **Sig. (p<0.001)** | | | |
| OT Area | 0.00000* |  |  |
| OT Roundness | 0.00000* |  |  |
| TH | 0.00000* |  |  |
| TL | 0.00000* |  |  |
| GW | 0.00000* |  |  |
| ABW | 0.00000* |  |  |
| PBW | 0.00000* |  |  |
| ISW | 0.28550 |  |  |
| SC Area | 0.00000* |  |  |
| SC Roundness | 0.00000* |  |  |
| WLM1 | 0.37639 |  |  |
| WLM2 | 0.62611 |  |  |
| WLM3 | 0.57442 |  |  |
| WLM4 | 0.17608 |  |  |
| WLM5 | 0.00000* |  |  |
| WLM6 | 0.00000* |  |  |
| WLM7 | 0.00000* |  |  |
| WLM8 | 0.00000* |  |  |
| WLM9 | 0.00000* |  |  |
| WLM10 | 0.00000* |  |  |
| WLM11 | 0.00000* |  |  |
| WLM12 | 0.00000* |  |  |
| WLM13 | 0.00000* |  |  |
| WLM14 | 0.00000* |  |  |
| WLM15 | 0.00000* |  |  |
| WLM16 | 0.00000* |  |  |
| WLM17 | 0.00000* |  |  |
| WLM18 | 0.00000* |  |  |
| WLM19 | 0.00000* |  |  |
| WLM20 | 0.00000* |  |  |
| WLM21 | 0.00000* |  |  |
| WLM22 | 0.00000* |  |  |
| WLM23 | 0.00004* |  |  |
| WLM24 | 0.03973 |  |  |
| WLM25 | 0.14531 |  |  |
| WLM26 | 0.00029* |  |  |
| WLM27 | 0.00006* |  |  |
| WLM28 | 0.00002* |  |  |
| WLM29 | 0.00011* |  |  |
| APC | 0.00000* |  |  |
| ALM-APC | 0.00003* |  |  |
| ILM5-APC | 0.00001* |  |  |
| ILM10-APC | 0.00000* |  |  |
| ILM15-APC | 0.00000* |  |  |
| ILM20-APC | 0.00000* |  |  |
| ILM25-APC | 0.00000* |  |  |
| PLM-APC | 0.00000* |  |  |
| SLM5-APC | 0.00000* |  |  |
| SLM10-APC | 0.00000* |  |  |
| SLM15-APC | 0.00000* |  |  |
| SLM20-APC | 0.00000* |  |  |
| SLM25-APC | 0.00000* |  |  |
| ANGLE | 0.00006* |  |  |

*Statistically significant.

A8: t-test bivariate comparison between the sample groups

| **Measurement Parameter** | **CT vs MRI** | **CT vs CAD** | **CAD vs MRI** |
| --- | --- | --- | --- |
| **Sig. (p<0.001)** | | | |
| OT Area | 0.00000* | 0.00000* | 0.04258 |
| OT Roundness | 0.00000* | 0.74809 | 0.00000* |
| TH | 0.00000* | 0.00160 | 0.00709 |
| TL | 0.00089* | 0.00000* | 0.00004* |
| GW | 0.00000* | 0.25173 | 0.00005* |
| ABW | 0.00000* | 0.11338 | 0.00000* |
| PBW | 0.00000* | 0.00002* | 0.00000* |
| ISW | 0.05562 | 0.30068 | 0.54870 |
| SC Area | 0.00000* | 0.00000* | 0.74908 |
| SC Roundness | 0.00000* | 0.90477 | 0.00000* |
| WLM1 | 0.50579 | 0.58818 | 0.18617 |
| WLM2 | 0.91806 | 0.65869 | 0.58873 |
| WLM3 | 0.57998 | 0.70467 | 0.94670 |
| WLM4 | 0.03478 | 0.71716 | 0.05510 |
| WLM5 | 0.00000* | 0.23796 | 0.00011* |
| WLM6 | 0.00000* | 0.87841 | 0.00000* |
| WLM7 | 0.00000* | 0.14732 | 0.00005* |
| WLM8 | 0.00000* | 0.00931 | 0.00031* |
| WLM9 | 0.00000* | 0.01643 | 0.00019* |
| WLM10 | 0.00000* | 0.06317 | 0.00000* |
| WLM11 | 0.00000* | 0.10534 | 0.00000* |
| WLM12 | 0.00000* | 0.23476 | 0.00000* |
| WLM13 | 0.00000* | 0.28615 | 0.00000* |
| WLM14 | 0.00000* | 0.87762 | 0.00000* |
| WLM15 | 0.00000* | 0.99615 | 0.00000* |
| WLM16 | 0.00000* | 0.10460 | 0.00002* |
| WLM17 | 0.00000* | 0.00865 | 0.00001* |
| WLM18 | 0.00000* | 0.00253 | 0.00000* |
| WLM19 | 0.00000* | 0.00007* | 0.00000* |
| WLM20 | 0.00000* | 0.00003* | 0.00000* |
| WLM21 | 0.00000* | 0.00061* | 0.00000* |
| WLM22 | 0.00000* | 0.00035* | 0.00007* |
| WLM23 | 0.00000* | 0.02304 | 0.05273 |
| WLM24 | 0.00533 | 0.23381 | 0.21740 |
| WLM25 | 0.01832 | 0.11785 | 0.79821 |
| WLM26 | 0.00006* | 0.00064* | 0.48448 |
| WLM27 | 0.00002* | 0.00039* | 0.61627 |
| WLM28 | 0.00002* | 0.00037* | 0.98387 |
| WLM29 | 0.00009* | 0.00951 | 0.45572 |

*Statistically significant.

A9: Hotelling’s T-squared test of OPA shape comparisons between MRI sample group and mental disorders of the CT sample group

| **Reference Group CC Shape** | **Compared Group CC Shape** | **CC Anatomical Region** | **HotT2-pval** | **Adj_p_x3** | **Adj_p_x6** |
| --- | --- | --- | --- | --- | --- |
| **Sig. (p<0.05)** | | | | | |
| MRI | Bipolar | Genu-Rostrum Region | 0.703527 | 1 | 1 |
| MRI | Schizophrenia | Genu-Rostrum Region | 0.780422 | 1 | 1 |
| MRI | Bipolar | Anterior body Region | 0.437196 | 1 | 1 |
| MRI | Schizophrenia | Anterior body Region | 0.027211* | 0.081634 | 0.163267 |
| MRI | Bipolar | Mid-body Region | 0.374162 | 1 | 1 |
| MRI | Schizophrenia | Mid-body Region | 0.103573 | 0.310718 | 0.621436 |
| MRI | Bipolar | Posterior body Region | 0.999159 | 1 | 1 |
| MRI | Schizophrenia | Posterior body Region | 0.977937 | 1 | 1 |
| MRI | Bipolar | Isthmus Region | 0.009398* | 0.028195* | 0.056391 |
| MRI | Schizophrenia | Isthmus Region | 0.001307* | 0.003921* | 0.007841* |
| MRI | Bipolar | Splenium Region | 0.959355 | 1 | 1 |
| MRI | Schizophrenia | Splenium Region | 0.991685 | 1 | 1 |

*Statistically significant.
